# Supplementary material for: Kaposi’s sarcoma-associated herpesvirus induces specialised ribosomes to efficiently translate viral lytic mRNAs
Source: Nat Commun. 2023 Jan 18;14:300. doi: 10.1038/s41467-023-35914-5 (PMC9849454; doi:10.1038/s41467-023-35914-5)
Supplement: Supplementary file 1 — Supplementary Information [file 41467_2023_35914_MOESM1_ESM.pdf]

Kaposi's sarcoma-associated herpesvirus induces specialised ribosomes to efficiently translate viral lytic mRNAs

James C. Murphy, Elena M. Harrington, Sophie Schumann, Elton J. R. Vasconcelos, Timothy J. Mottram, Katherine L. Harper, Julie L. Aspden and Adrian Whitehouse

Supplementary Figures 1-22 and Legends

Supplementary Tables 1-3

DIMT1

a

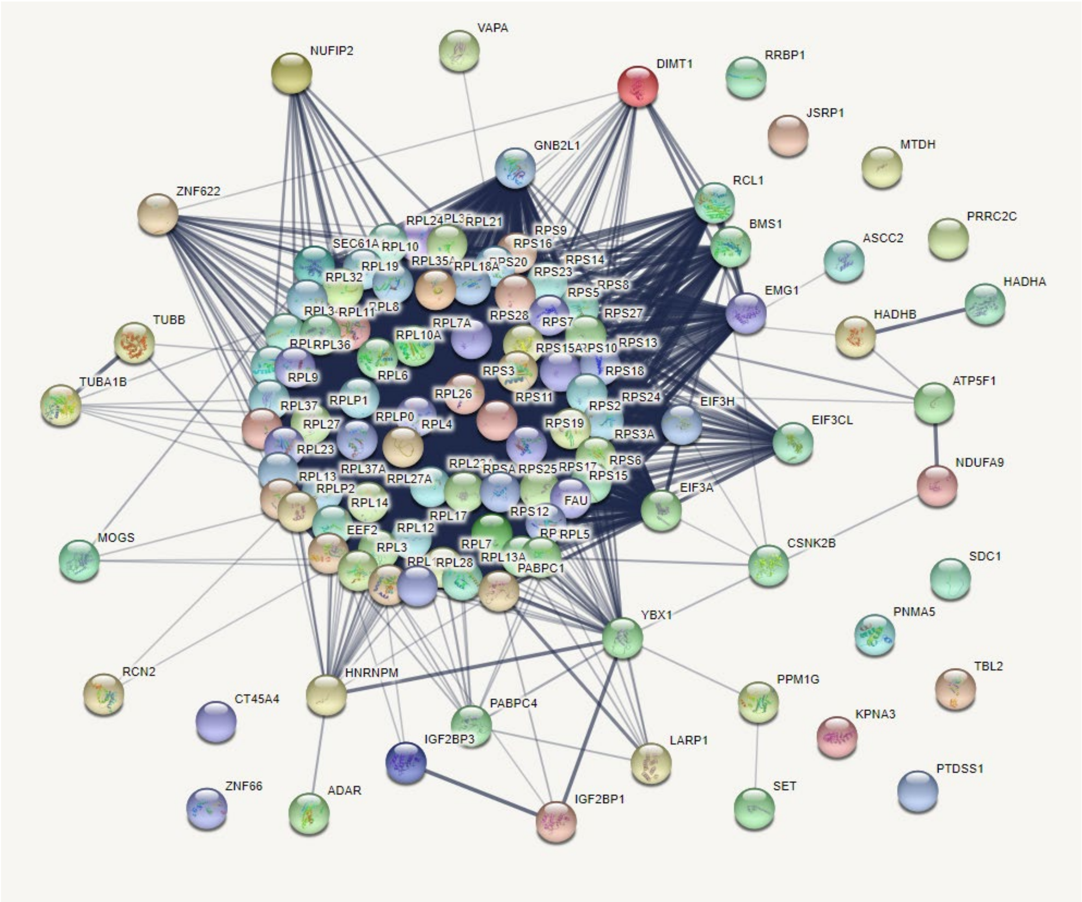

b

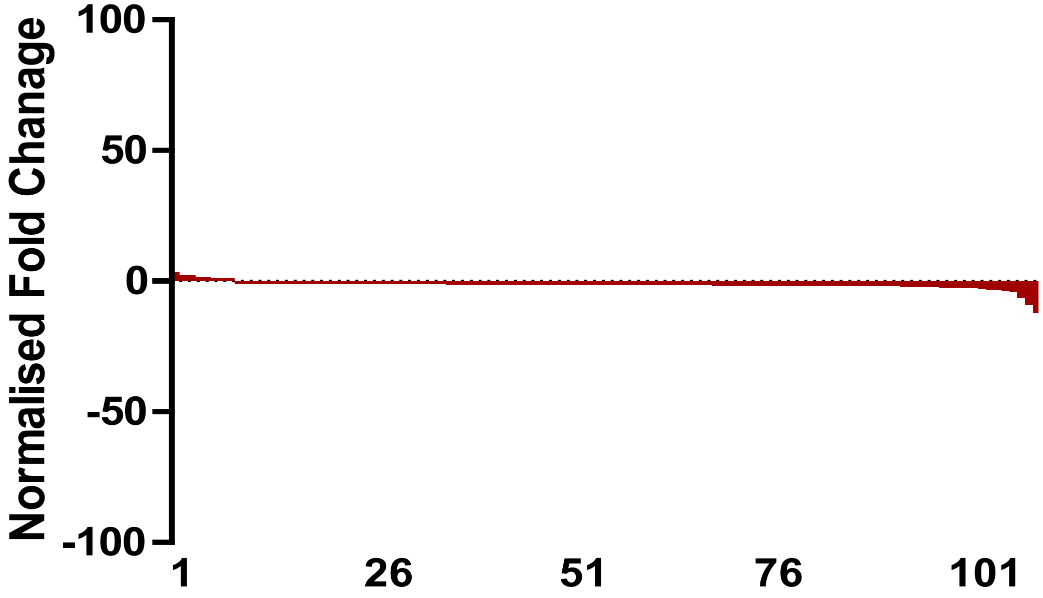

Supplementary Figure 1. TMT LC-MS/MS analysis of DIMT1 Twin-Strep-tag® pulldowns from a latent and 24 hour post lytic reactivation TREx BCBL1-Rta cell line. STRING protein interaction map of identified proteins **(a)**. Fold change of all interacting proteins from latent compared to 24 hours post lytic reactivation **(b)**.

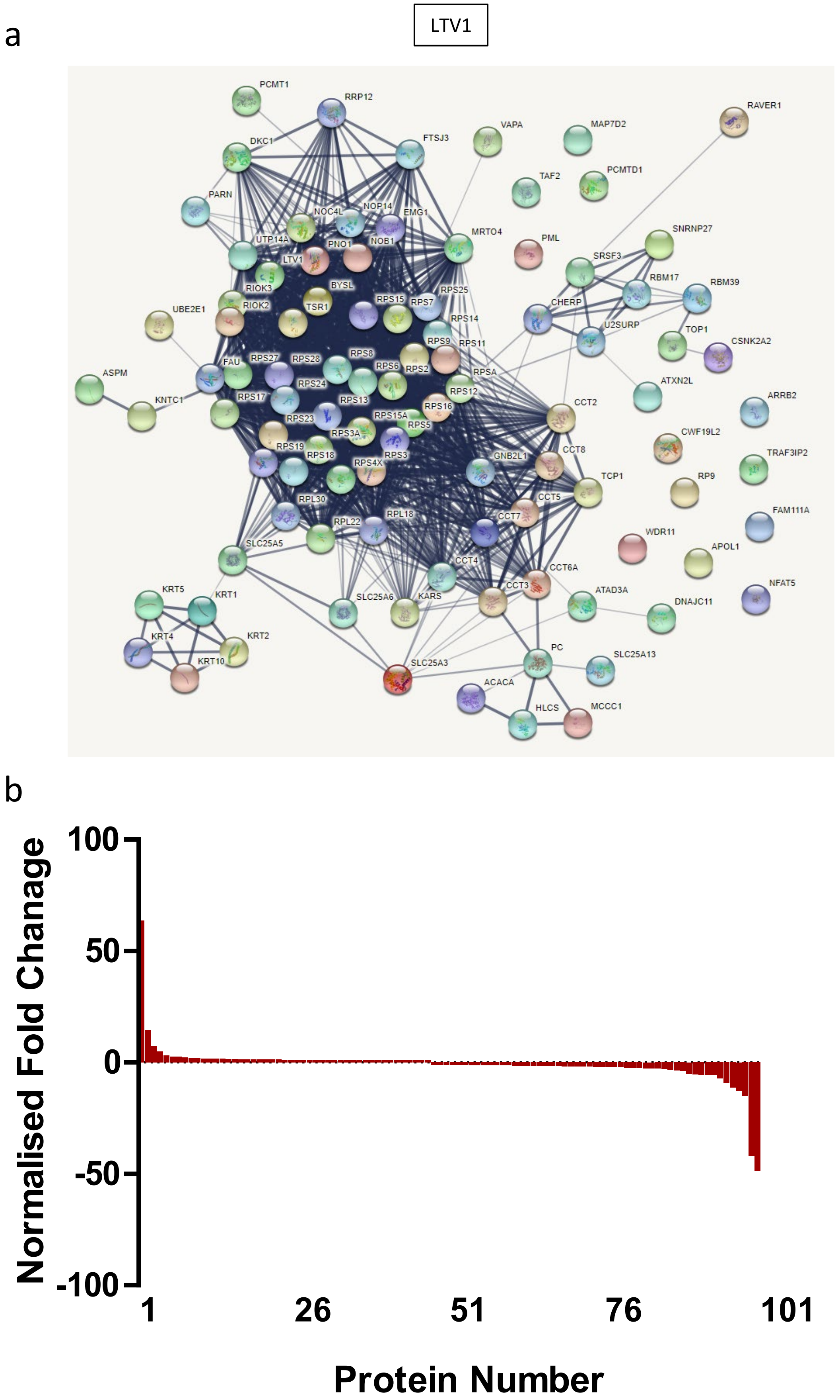

Supplementary Figure 2. TMT LC-MS/MS analysis of LTV1 Twin-Strep-tag® pulldowns from a latent and 24 hour post lytic reactivation TREx BCBL1-Rta cell line. STRING protein interaction map of identified proteins **(a)**. Fold change of all interacting proteins from latent compared to 24 hours post lytic reactivation **(b)**.

TSR1

a

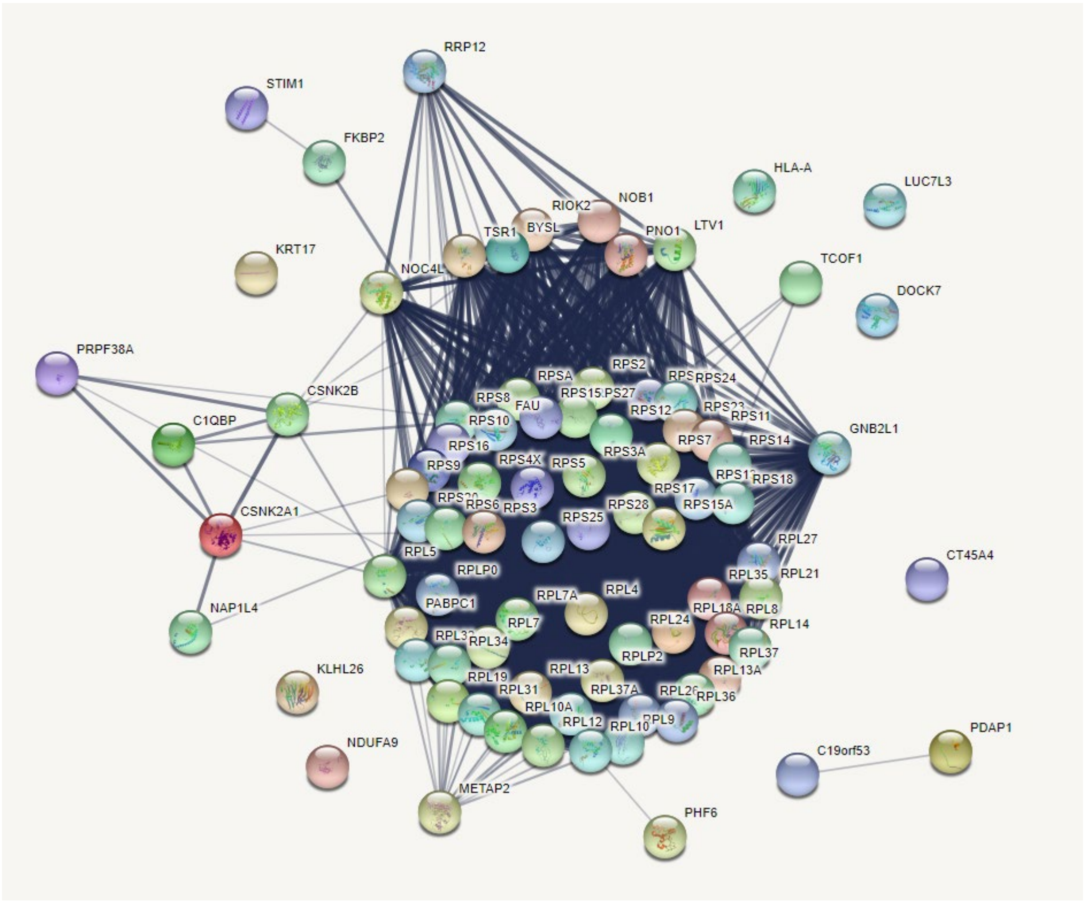

b

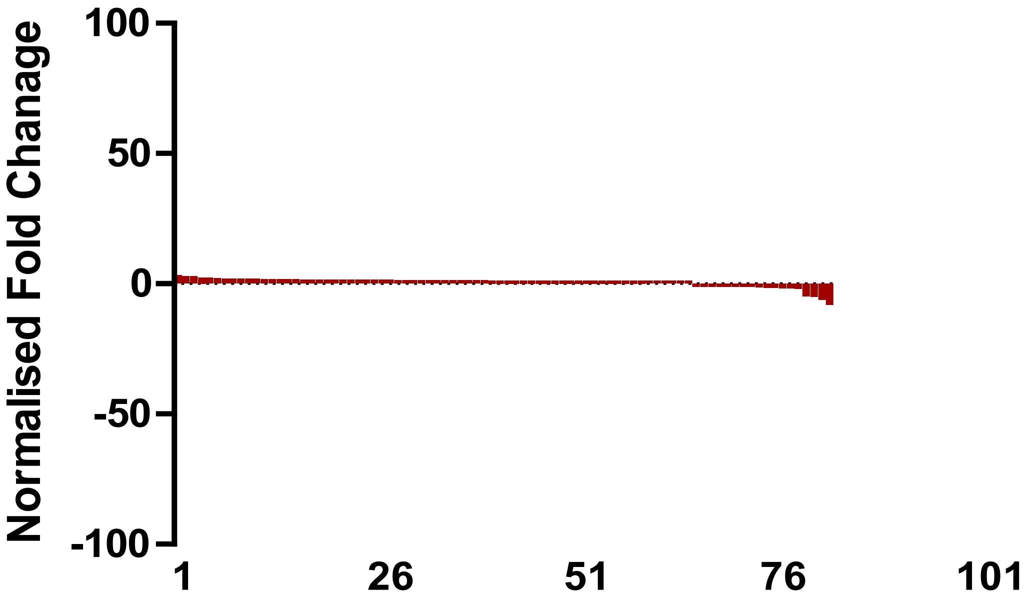

Supplementary Figure 3. TMT LC-MS/MS analysis of TSR1 Twin-Strep-tag® pulldowns from a latent and 24 hour post lytic reactivation TREx BCBL1-Rta cell line. STRING protein interaction map of identified proteins **(a)**. Fold change of all interacting proteins from latent compared to 24 hours post lytic reactivation **(b)**.

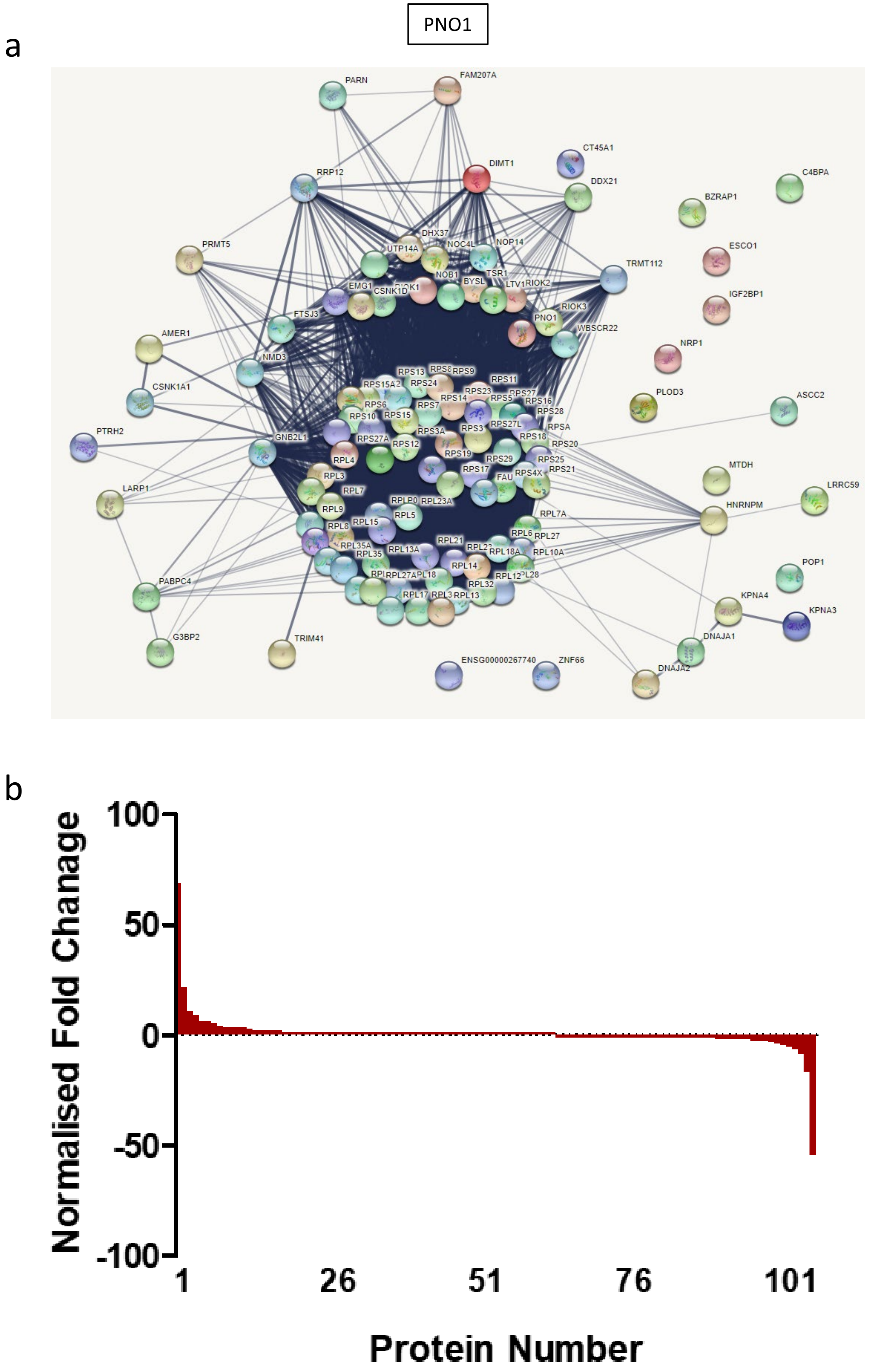

Supplementary Figure 4. TMT LC-MS/MS analysis of PNO1 Twin-Strep-tag® pulldowns from a latent and 24 hour post lytic reactivation TREx BCBL1-Rta cell line. STRING protein interaction map of identified proteins **(a)**. Fold change of all interacting proteins from latent compared to 24 hours post lytic reactivation **(b)**.

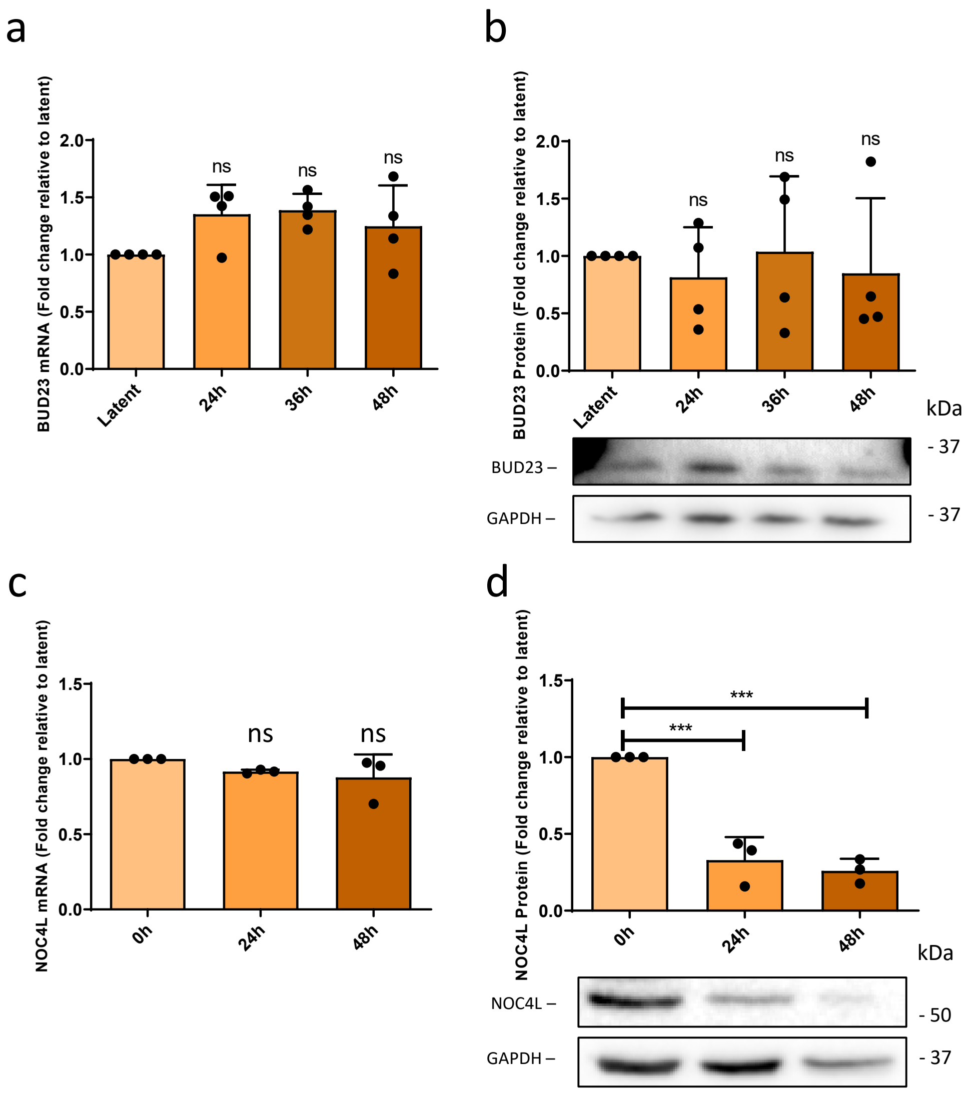

Supplementary Figure 5. BUD23 and NOC4L expression during lytic reactivation of KSHV in TREx BCBL1-Rta cells. Whole cell lysates were collected at various timepoints after lytic reactivation and split in two. Total RNA was isolated from half the cell lysates and BUD23 or NOC4L mRNA production was assayed by two step RT-qPCR and analysed by comparison to the 0h (latent) control using a ΔΔCt method (n=3 biologically independent samples) **(a,c)**. The second half of the whole cell lysates were analysed by western blot probing for BUD23 or NOC4L, GAPDH was included as a reference gene, representative western blots and densitometric analysis relative to the Scr control (n=3 biologically independent samples) (p=0.0003 and 0.0002) **(b,d)**. Data are presented as mean ± SD. Significance was calculated by one-way ANOVA with a Newman-Keuls multiple comparison post-test. Asterisks denote a significant difference between the specified groups (* p≤ 0.05, ** p<0.01 and *** p<0.001). NS = Not significant.

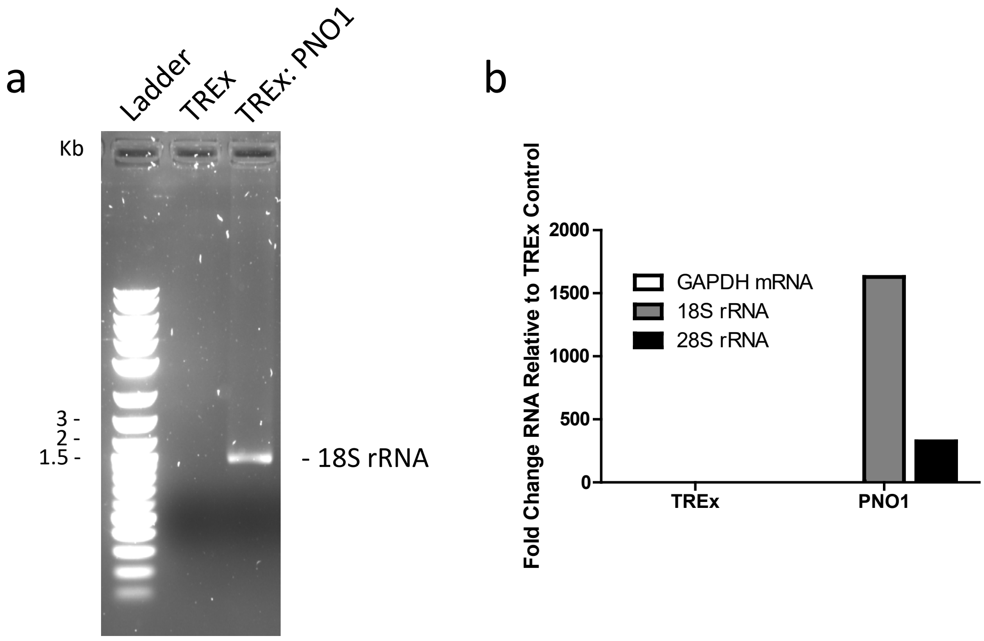

Supplementary Figure 6. The RNA composition of isolated pre-40S complexes by PNO1 Twin-Strep-tag® pulldowns. Total nucleic acids was isolated from whole cell lysate pulldowns from control cells and PNO1 bait protein expressing cells. Denaturing polyacrylamide gel electrophoresis **(a)** and two-step RT-qPCR, with primers specific for GAPDH mRNA, 18S rRNA and 28S rRNA **(b)**.

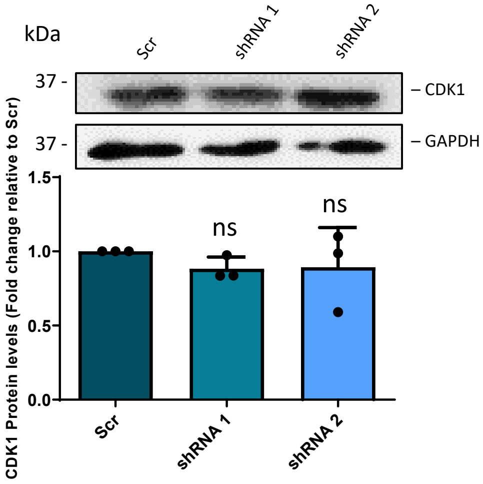

Supplementary Figure 7. BUD23 depletion does not impact protein turnover. Whole cell lysates were collected form TREx BCBL1-Rta cells expressing a Scr shRNA or two different shRNAs targeting BUD23. Lysates were analysed by western blot probing for CDK1 and GAPDH was included as a reference gene, representative western blots and densitometric analysis relative to the Scr control (n=3 biologically independent samples). Data are presented as mean ± SD. Significance was calculated by one-way analysis of variance (ANOVA) with a Newman-Keuls multiple comparison post-test, ns = not significant.

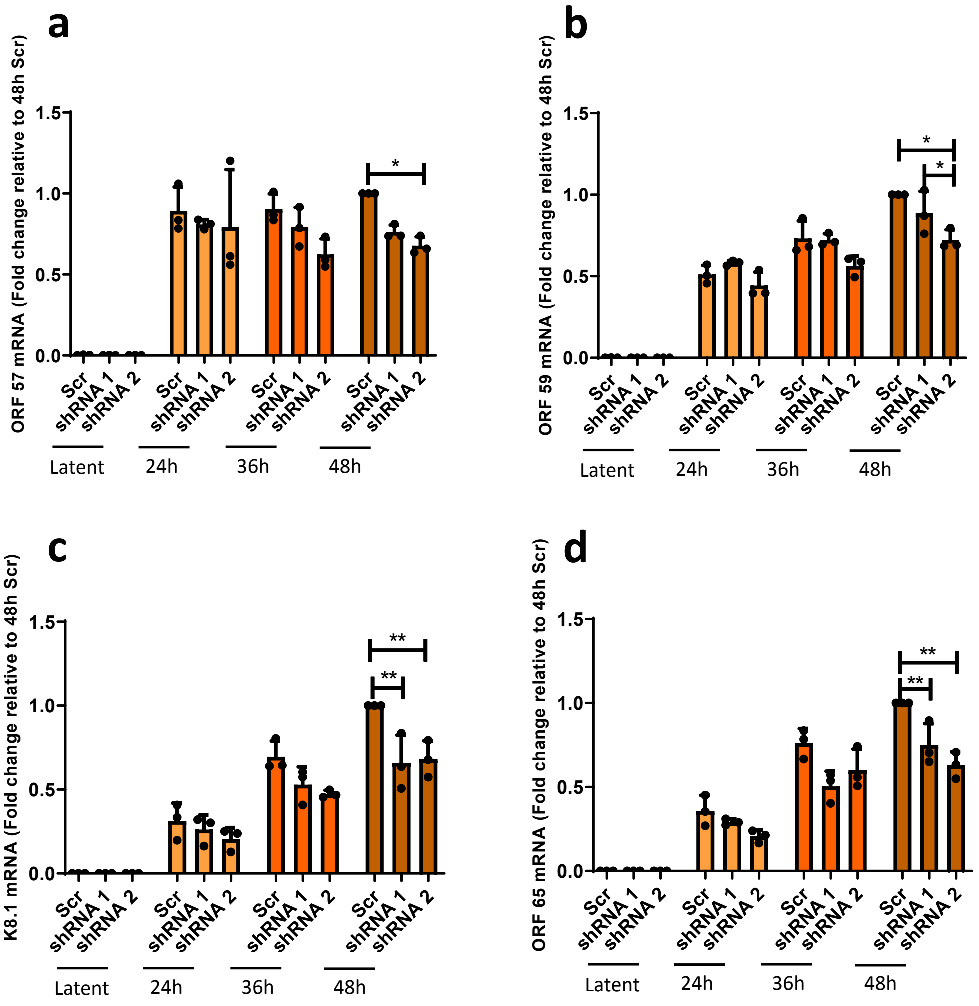

Supplementary Figure 8. Depletion of BUD23 has a minor affect on transcription of KSHV lytic mRNAs. TREx BCBL1-Rta cells expressing a Scr shRNA or two different shRNAs targeting BUD23 were used for a time course of KSHV lytic reactivation over 48 hours, with samples collected at 0 (latent), 24, 36 and 48 hours (n=3 biologically independent samples). Total RNA was isolated from cells and quantified by two-step RT-qPCR, with primers specific for viral genes, ORF57 **(a)**, ORF59 **(b)**, K8.1 **(c)**, and ORF65 **(d)**, and human GAPDH as a quantity control. Data was analysed by comparison to GAPDH and the 48h Scr control using a ΔΔCt method. Data are presented as mean ± SD. Significance was calculated by one-way ANOVA with a Newman-Keuls multiple comparison post-test. Asterisks denote a significant difference between the specified groups (* $p \leq 0.05$, ** $p<0.01$ and *** $p<0.001$).

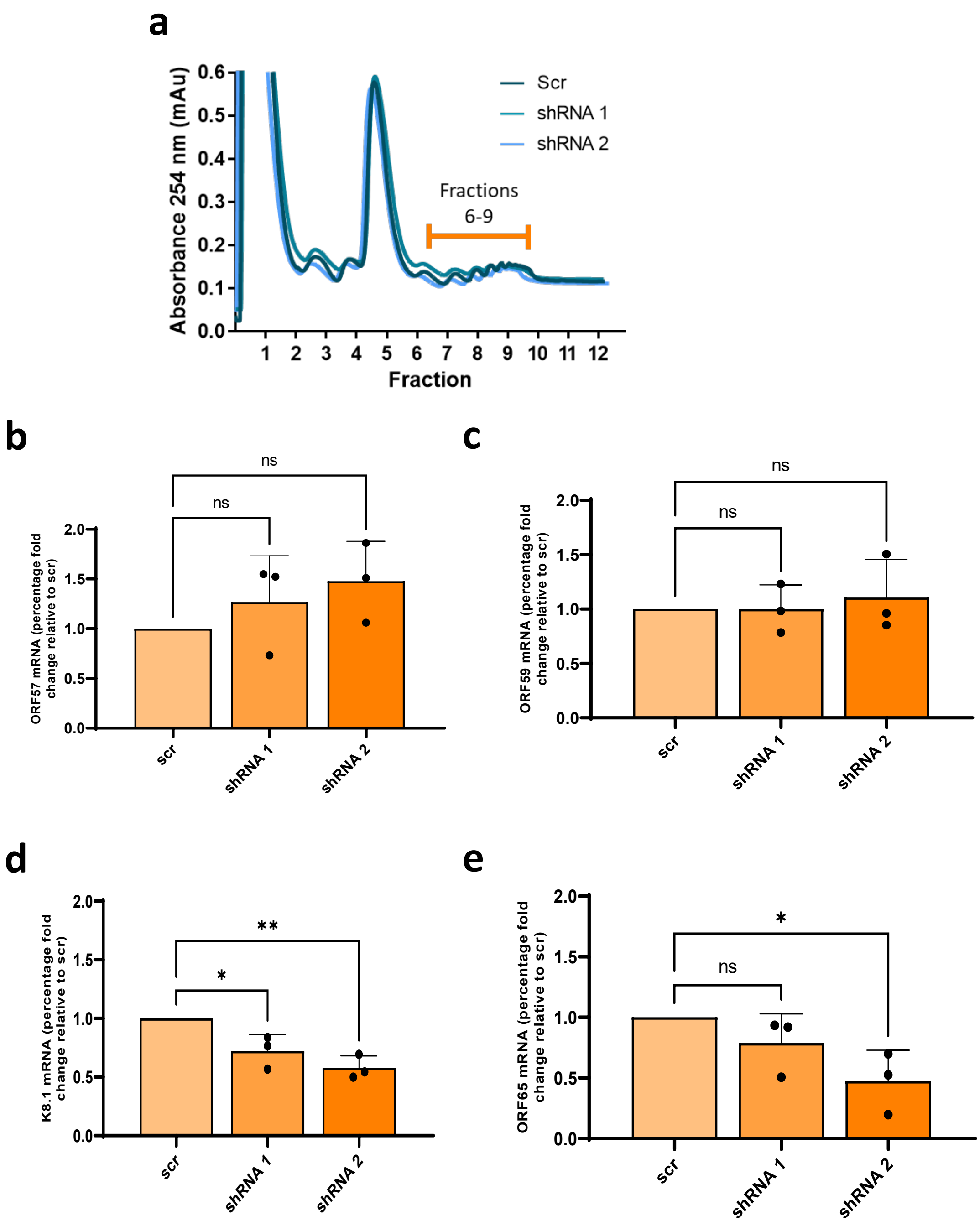

Supplementary Figure 9. Depletion of BUD23 reduces translation of late lytic KSHV mRNAs but not early mRNAs. KSHV lytic replication was induced for 36 hours in TREx BCBL1-Rta cells expressing a Scr shRNA or two different shRNAs targeting BUD23 (n=3 biologically independent samples). Polysome profiling was performed and the polysome fractions (6-9) were collected **(a)** and total RNA was isolated and quantified by two-step RT-qPCR, with primers specific for viral genes, ORF57 **(b)**, ORF59 **(c)**, K8.1 (p = 0.030 and 0.0051) **(d)**, and ORF65 (p= 0.0443) **(e)**, and human GAPDH as a quantity control. Data was analysed by comparison to GAPDH and the Scr control relative to total mRNA input using a ΔΔCt method. Data are presented as mean ± SD. Significance was calculated by one-way ANOVA with a Tukey's multiple comparison post-test. Asterisks denote a significant difference between the specified groups (* p≤ 0.05, ** p<0.01 and *** p<0.001).

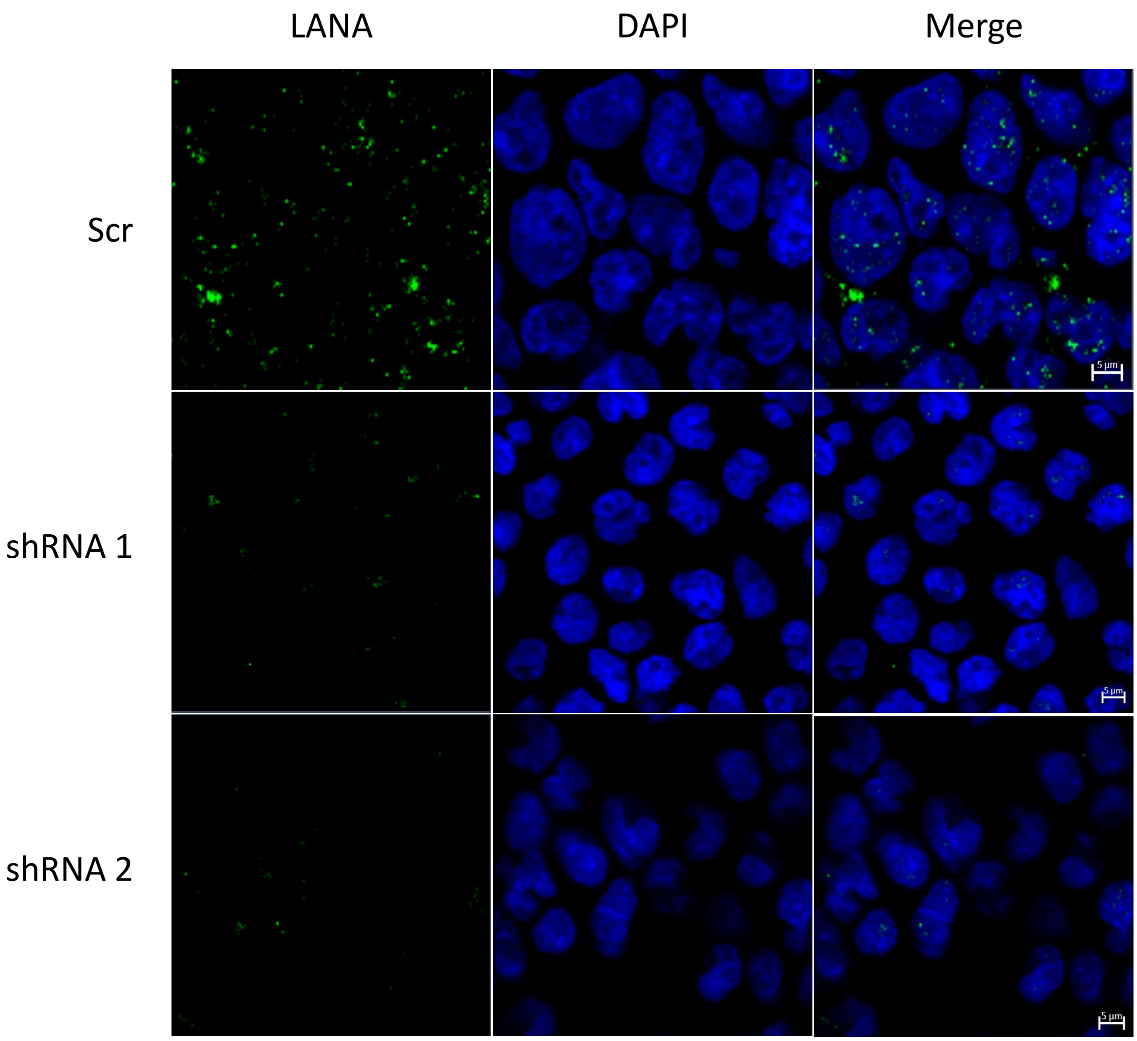

Supplementary Figure 10. Depletion of BUD23 dramatically reduces infectious virion production for re-infection of naive HEK-293T cells. Lytic reactivation of KSHV was induced in TREx BCBL1-Rta cells expressing a Scr shRNA or two different shRNAs targeting BUD23 for 72 hours. Virus released from TREx BCBL1-Rta cell lines was collected and HEK 293T cells re-infected with the virus for 48 hours. Cells were fixed, permeabilised, and stained for the KSHV viral protein LANA (green) and the DNA dye DAPI (blue), then mounted and viewed using an LSM 880 inverted confocal microscope. Representative images are shown (n=3 biologically independent samples).

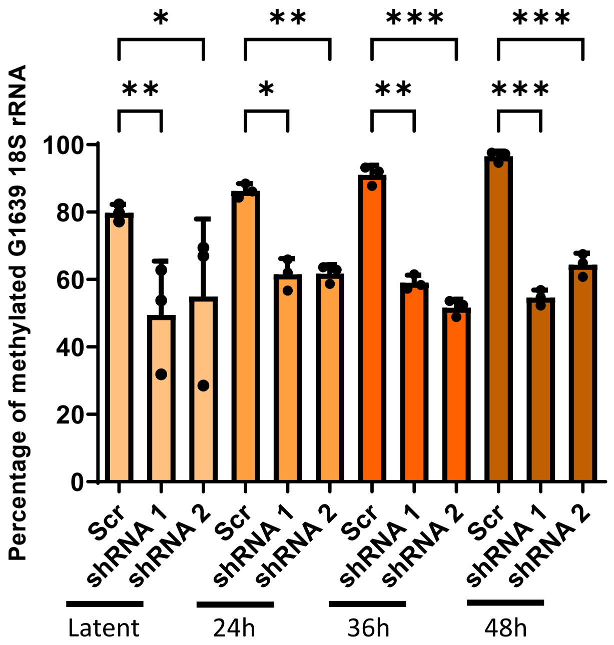

Supplementary Figure 11. Depletion of BUD23 reduces N^7-methylation of the 18S rRNA base G1639 throughout KSHV lytic replication. TREx BCBL1-Rta cells expressing a Scr shRNA or two different shRNAs targeting BUD23 were used for a time course of KSHV lytic reactivation over 48 hours, with samples collected at 0 (latent), 24, 36 and 48 hours (n=3). Total RNA was isolated and chemical cleaved at m7G sites. The proportion of cleaved to uncleaved rRNA at 18S G1639 was determined by qPCR with primers flanking the cleave site and analysed using a ΔΔCt method (n=3 biologically independent samples). Data are presented as mean ± SD. Significance was calculated by one-way ANOVA with a Newman-Keuls multiple comparison post-test. Asterisks denote a significant difference between the specified groups (* p≤ 0.05, ** p<0.01 and *** p<0.001).

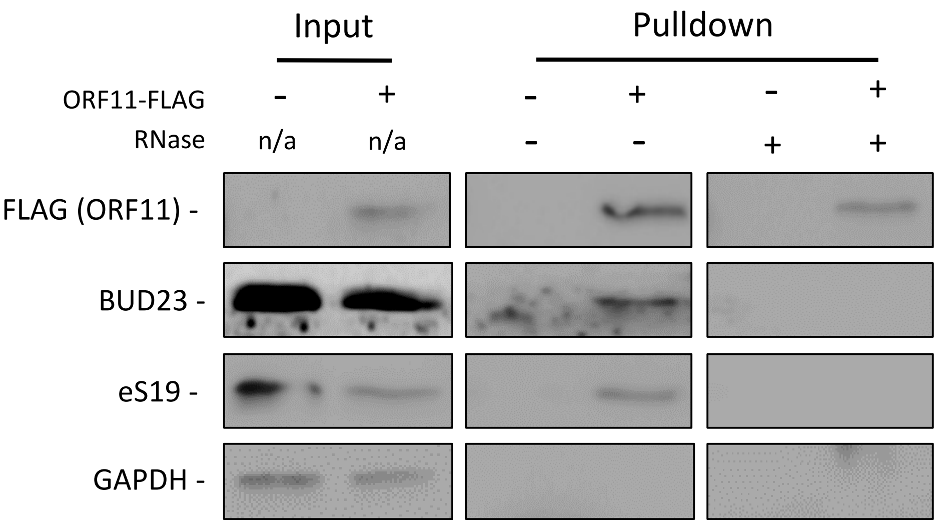

Supplementary Figure 12. The interaction of ORF11 with BUD23 is dependent on the 18S rRNA. Whole cell lysates from TREx BCBL1-Rta cells or TREx BCBL1-Rta cells expressing a ORF11-FLAG were subject to FLAG pulldowns in the presence and absence of RNase A, whole cell lysate input references where also taken. Samples where analysed by western blot probing for FLAG (ORF11), BUD23, eS19 and GAPDH, representative western blots are shown (n=3 biologically independent samples).

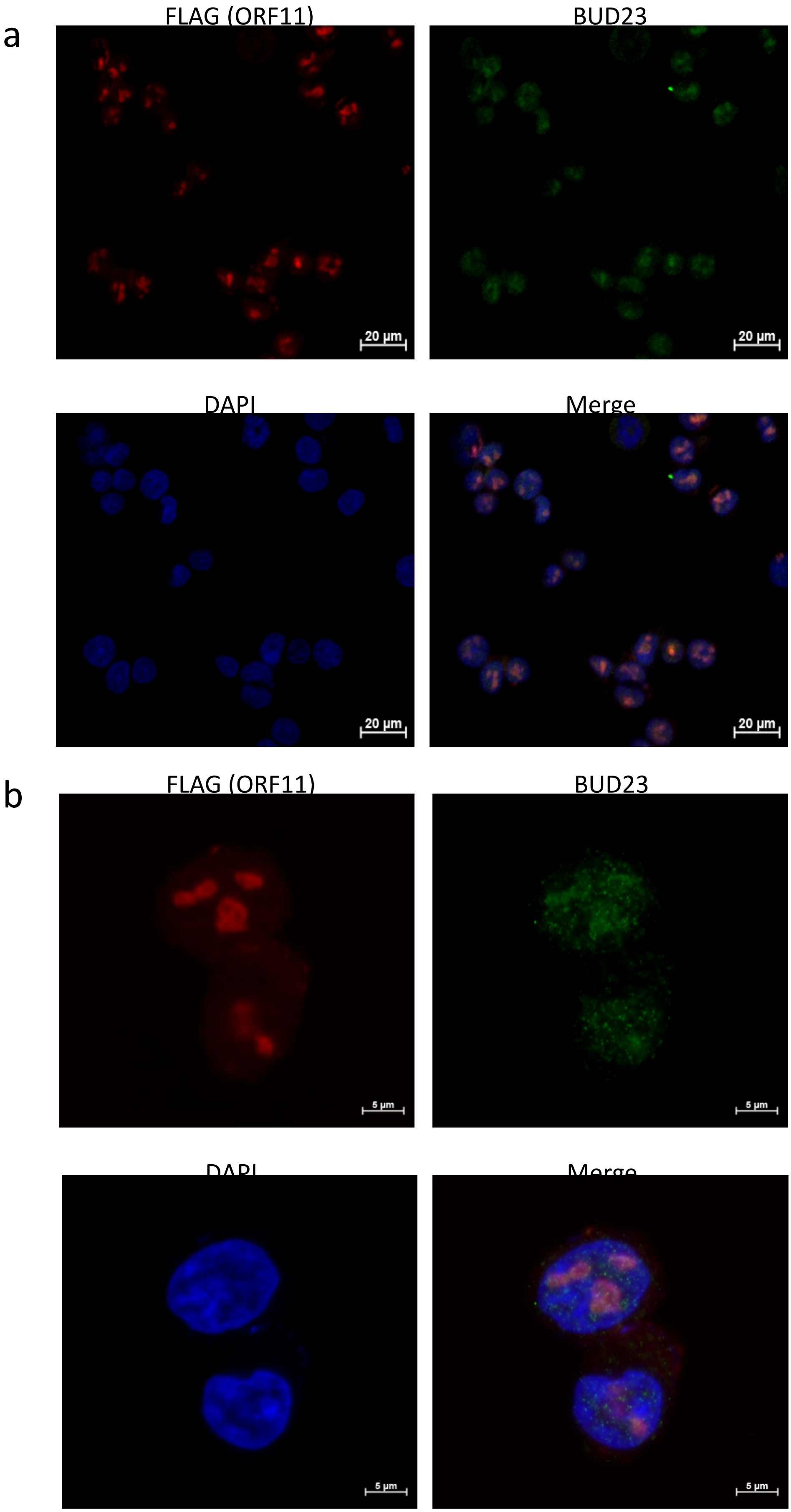

Supplementary Figure 13. Immunofluorescence microscopy demonstrates KSHV protein ORF11 localises mainly to the nucleolus and nucleoplasm. KSHV lytic reactivation was induced for 16 hours in TREx BCBL1-Rta cells stably expressing ORF11-FLAG and were fixed, permeabilised, and stained for FLAG (red), BUD23 (Green) and the DNA dye DAPI (blue). The cells were mounted and viewed using an LSM 880 inverted confocal microscope **(a,b)**. Representative images are shown (n=3 biologically independent samples).

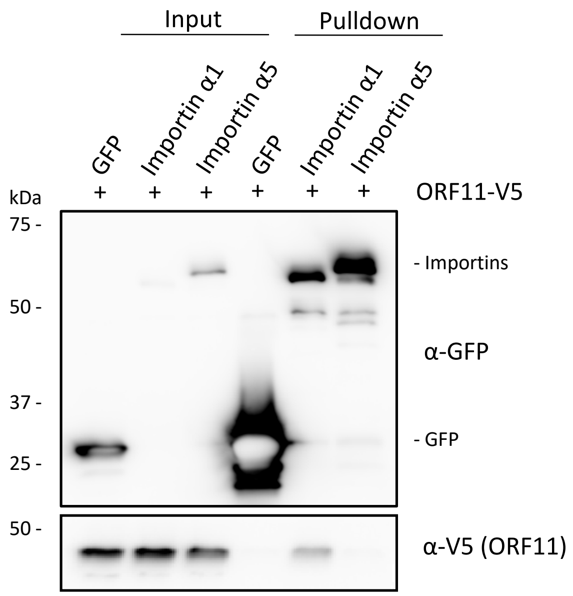

Supplementary Figure 14. The KSHV protein ORF11 interacts with importin α1. GFP-Trap® pulldowns of HEK 293T whole cell lysates. All cells were transfected with a plasmid encoding for the expression of ORF11-V5 and another plasmid encoding either GFP, GFP-importin α1 or GFP-importin α5. Representative western blots are shown (n=3 biologically independent samples).

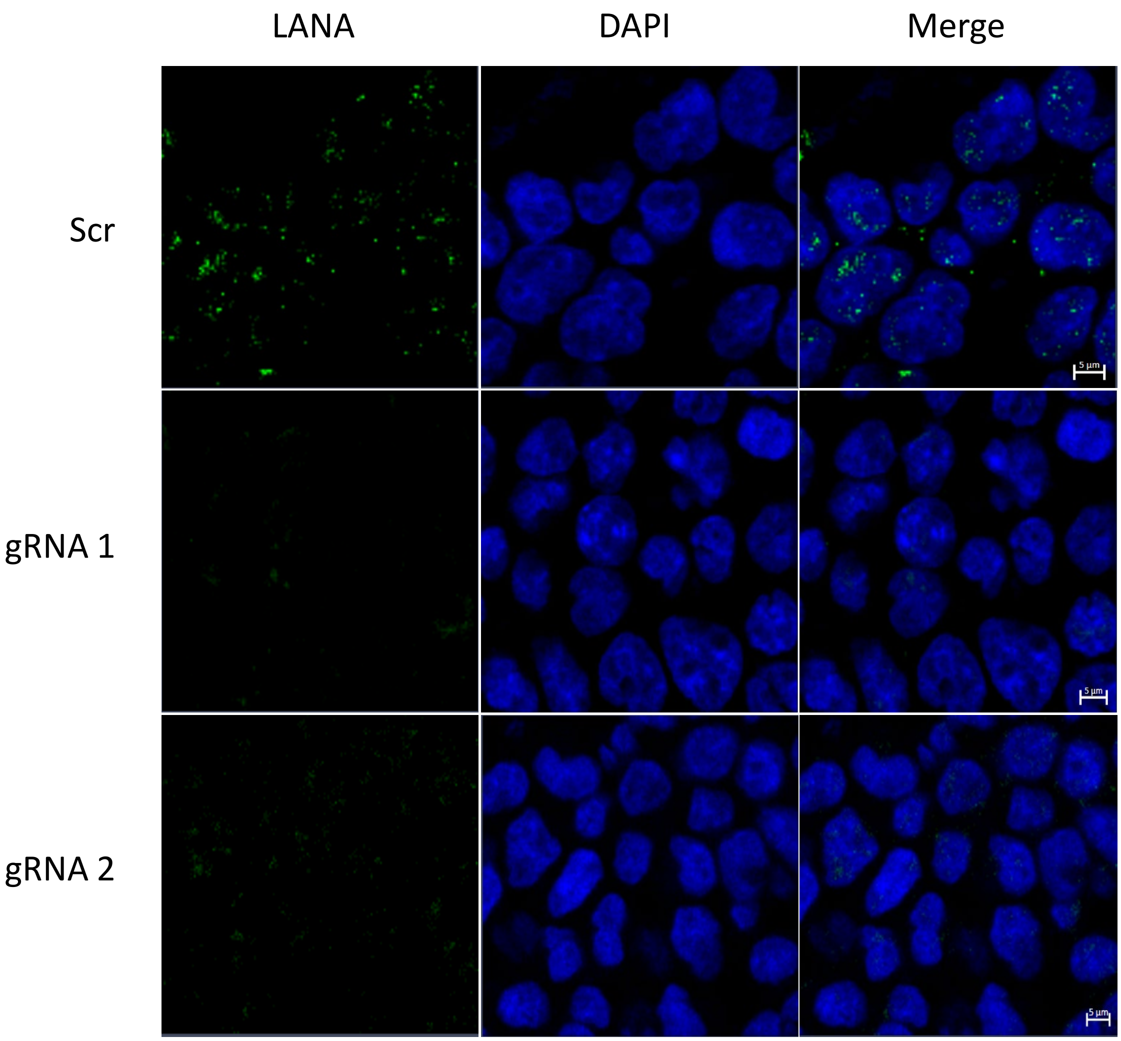

Supplementary Figure 15. Knockout of the KSHV protein ORF11 dramatically reduces infectious virion production for re-infection of naive HEK-293T cells. Lytic reactivation of KSHV was induced in TREx BCBL1-Rta cells expressing a Scr gRNA or two different gRNAs targeting ORF11 for 72 hours. Virus released from TREx BCBL1-Rta cell lines was collected and HEK 293T cells re-infected with the virus for 48 hours. Cells were fixed, permeabilised, and stained for the KSHV viral protein LANA (green) and the DNA dye DAPI (blue), then mounted and viewed using an LSM 880 inverted confocal microscope. Representative images are shown (n=3 biologically independent samples).

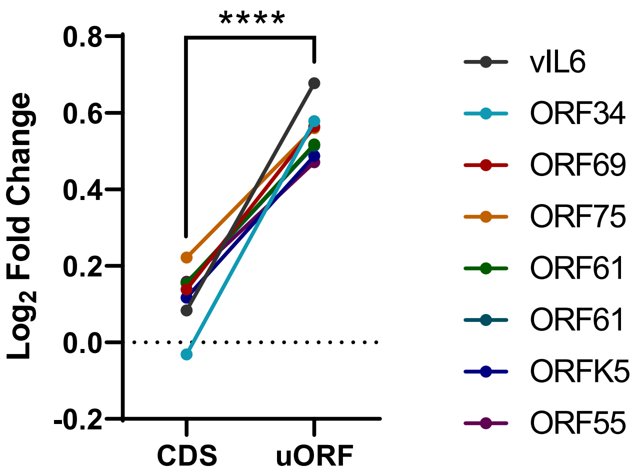

Supplementary Figure 16. uORFs with the greatest increase in translational efficiency, during BUD23 depletion, are significantly more translated than their corresponding CDS. Changes in translational efficiency of uORFs compared to their corresponding CDS (p = <0.0001). Significance was calculated by two-tailed paired T test, asterisks denote a significant difference between the specified groups (**** p<0.0001).

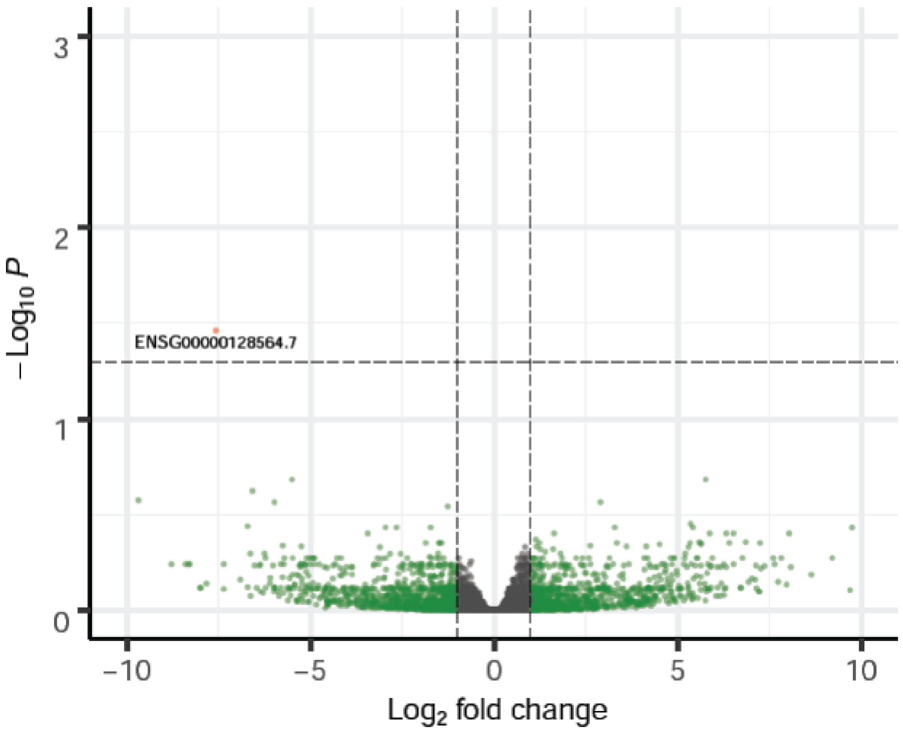

Supplementary Figure 17. Depletion of BUD23 does not significantly effect the translation of human transcripts. Changes in translational efficiency of human genes calculated for libraries originating from TREx BCBL1-Rta cells expressing a Scr shRNA compared to shRNA 1 targeting BUD23 at 36 hours post KSHV lytic reactivation. RiboRex Bioconductor package (PMID: 28158331) was used, relying on DESeq2 negative binomial model, for two-tailed differential translation (DT) calculations. A false discovery rate was set at < 0.005 and fold change threshold set at ± 0.45 (log2). Volcano plot representation of all KSHV gene translational efficiency changes (n=2 biologically independent samples).

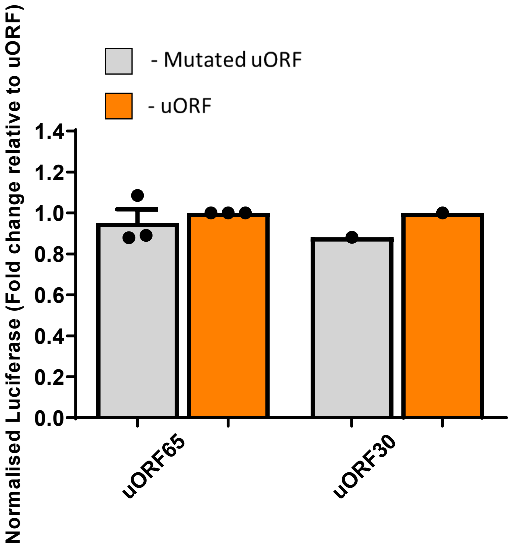

Supplementary Figure 18. Luciferase reporter assay of KSHV uORFs which do not effect the expression of the downstream luciferase. Normalised luciferase intensity was quantified from luciferase reported plasmids containing uORFs or with the start codon of the uORF mutated in HEK 293T cells (n=3 biologically independent samples). Data are presented as mean ± SD.

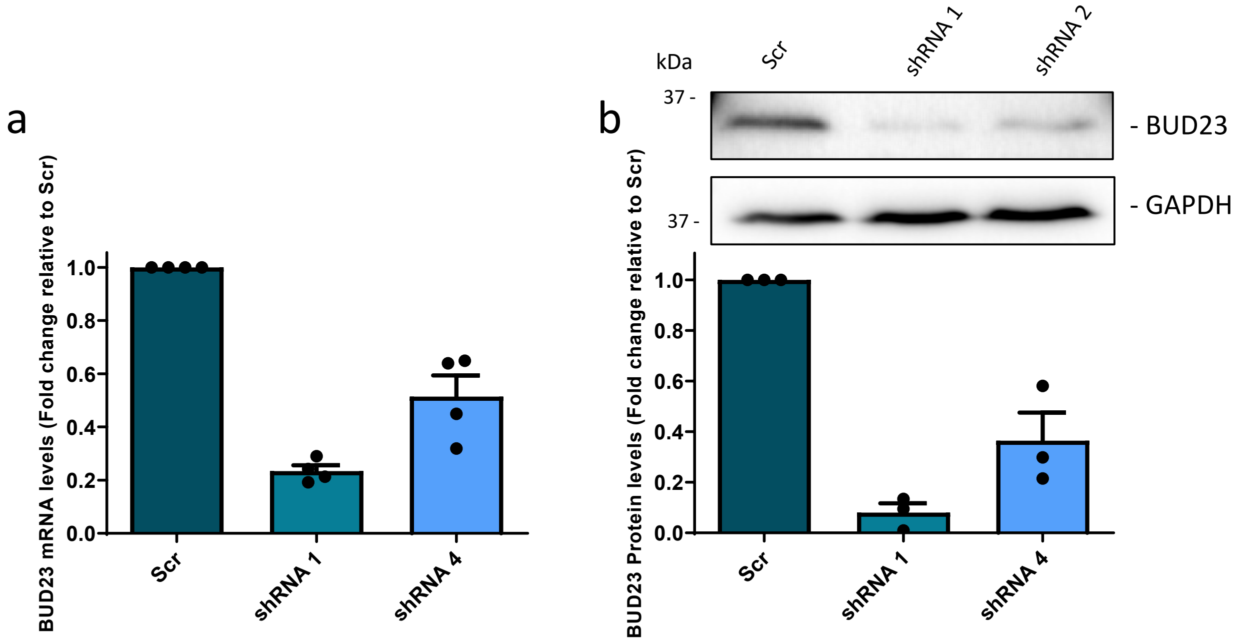

Supplementary Figure 19. Knockdown of BUD23 in HEK 293T cells. A Lentivirus expression system was used to stably transduced HEK 293T cells with a non-targeting scrambled shRNA (Scr) or two different shRNAs targeting BUD23 (shRNA 1 and 2). BUD23 mRNA production was assayed by two step RT-qPCR and analysed by comparison to the Scr control using a ΔΔCt method (n=4) **(a)**. Whole cell lysates were collected and analysed by western blot probing for BUD23, GAPDH was included as a reference gene, representative western blots and densitometric analysis relative to the Scr control (n=3 biologically independent samples). **(b)**. Data are presented as mean ± SD.

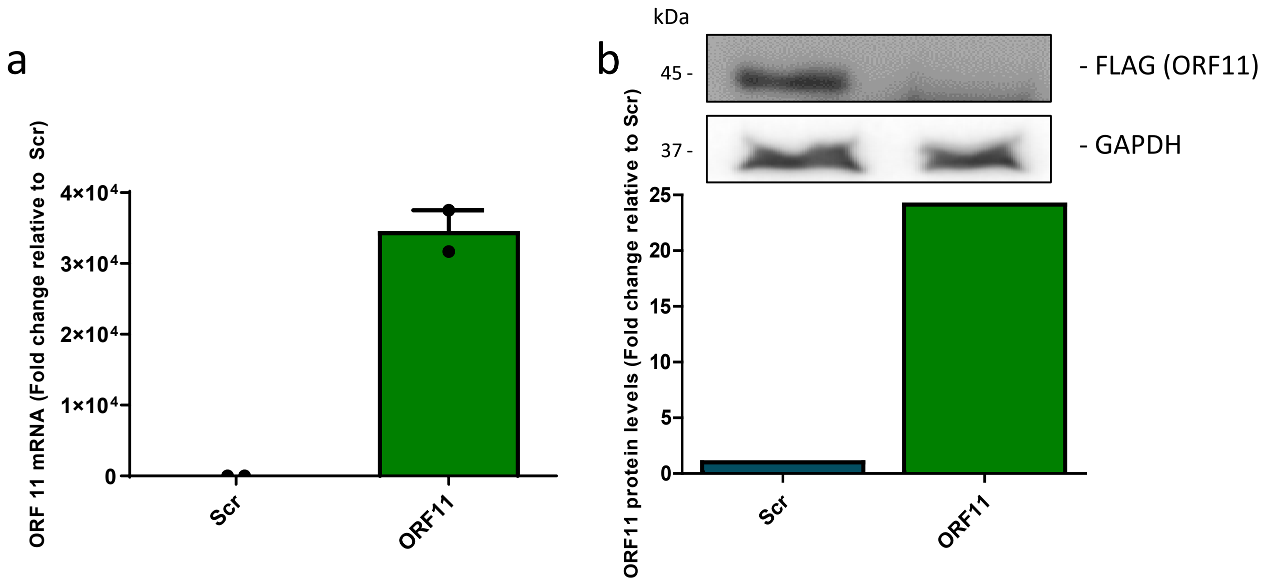

Supplementary Figure 20. Expression of ORF11-FLAG in HEK 293T cells. A Lentivirus expression system was used to stably transduced HEK 293T cells with a non-targeting scrambled shRNA (Scr) orORF11-FLAG expression plasmid. ORF11 mRNA production was assayed by two step RT-qPCR and analysed by comparison to the Scr control using a ΔΔCt method (n=2 biologically independent samples) **(a)**. Whole cell lysates were collected and analysed by western blot probing for FLAG, GAPDH was included as a reference gene, representative western blots and densitometric analysis relative to the Scr control (n=2 biologically independent samples) **(b)**. Data are presented as mean ± SD.

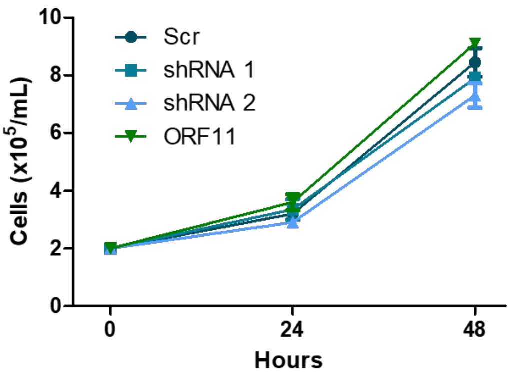

Supplementary Figure 21. Proliferation of HEK 293T cell lines. HEK 293T cell lines were counted over 48 hours to measure cell proliferation (n=2 biologically independent samples). Data are presented as mean ± SD.

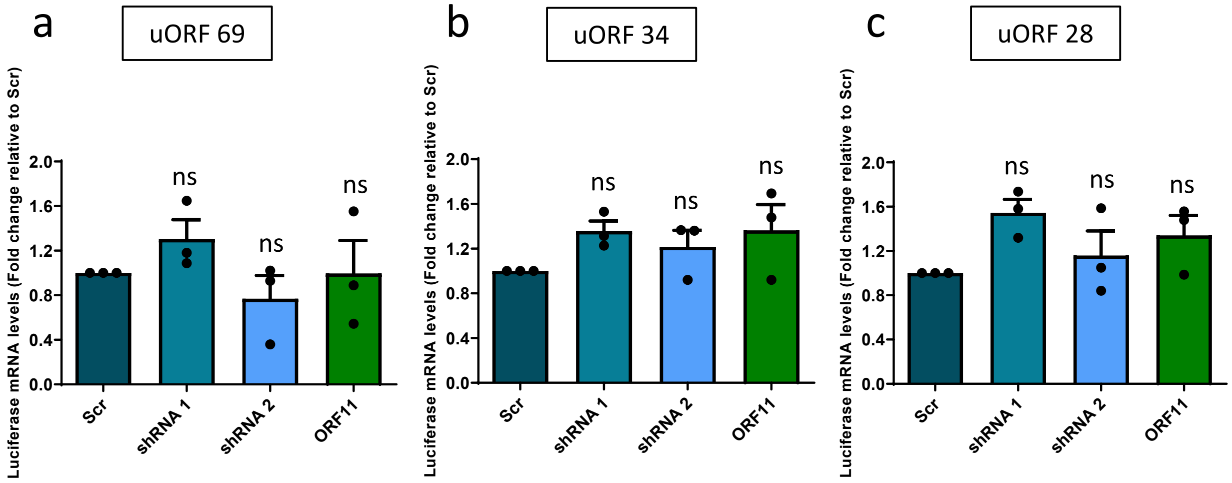

Supplementary Figure 22. The 5'UTR of ORF69, 34 and 28 do not affect the transcription of a gene during BUD23 depletion or with expression of ORF11. HEK 293T cells stabling expressing a non-targeting scrambled (Scr) shRNA or shRNAs 1 or 2 targeting BUD23 or ORF11-FLAG exogenous expression were transfected with luciferase reported plasmids containing uORFs 69 **(a)**, 34 **(b)** and 28 **(c)** (n=3 biologically independent samples). Total RNA was isolated from cells and quantified by two-step RT-qPCR, primers specific for renilla luciferase and firefly luciferase were used. Renilla luciferase levels were analysed by comparison to the transfection control firefly luciferase and the Scr control using a ΔΔCt method. Data are presented as mean ± SD. Significance was calculated by one-way ANOVA with a Newman-Keuls multiple comparison post-test. Asterisks denote a significant difference between the specified groups (ns = not significant).

Plasmid	Vector	Source
Lentiviral packaging	psPAX2	A gift from Dr. Edwin Chen (University of Leeds)
Lentiviral envelope	pVSV.G	
FLAG-2xStep-DIMT1	pCDH-CMV-MCS-EF1-Puro	GenScript (Custom made)
FLAG-2xStep-PNO1		
FLAG-2xStep-LTV1		
FLAG-2xStep-TSR1		
Scr shRNA	pLKO.1	Dharmacon (RHS4459)
BUD23 shRNA 1		Dharmacon (TRCN0000140056)
BUD23 shRNA 2		Dharmacon (TRCN0000142391)
GFP	pLenti-III-mir-GFP	Cloned by aurthors for this study.
GFP-ORF11		
ORF11-FLAG	pLenti-CMV-Puro	
ORF11-HA	pLenti-CMV-Zeo	
ORF11-V5	pcDNA™3.1/nV5-DEST	A gift from Prof. Yan Yuan (University of Pennsylvania)
GFP-importin α1	pcDNAGFP	Previously described[47].
GFP-importin α5		
Scr gRNA	lentiCRISPR V2	Cloned by aurthors for this study.
ORF11 gRNA 1		
ORF11 gRNA 2		
uORF69	psiCHECK™-2	Cloned by aurthors for this study.
uORF69 - Mutant		
uORF34		
uORF34 - Mutant		
uORF28		
uORF28 - Mutant		
uORF65		
uORF65 - Mutant		
uORF30		
uORF30 - Mutant		

Supplementary Table 1. All plasmids used in this study.

Primer	Forward	Reverse
GAPDH	TGT GGT CAT GAG TCC TTC CAC GAT	AGG GTC ATC ATC TCT GCC CCC TC
BUD23	TAC GTT CGC AAC TCA CGG AT	CCA GCA GGT AAC AGG GCT TA
18S rRNA total	GAT GGT AGT CGC CGT GCC	GCC TGC TGC CTT CTT TGG
18S rRNA m^{7}G 1639	GTA ACC CGT TGA ACC CCA TT	CCA TCC AAT CGG TAG TAG CG
ORF57	GCC ATA ATC AAG CGT ACT GG	GCA GAC AAA TAT TGC GGT GT
ORF59	CCG ATC GRG GAA AGG TAG GA	ATG TAC TCG ACG CTG GCA TA
K8.1	GTTCCACACAGATTCGCACA	AGTTCATCCTGCCTAGCCAG
ORF65	AAG GTG AGA GAC CCC GTG AT	TCC AGG GTA TTC ATG CGA GC
NOC4L	GCT TCT ATG TGA AGC GGG CG	GCC TGG AAA ACC CTC CTG TG
28S rRNA	GGG TGG TAA ACT CCA TCT AAG G	GCC CTC TTG AAC TCT CTC TTC
ORF11	TCC TCG AGC GTG CTG ATT TT	TAT TTT GAG CCC TCC CAC GG
Firefly luciferase	GCA GTT CTT CAT GCC AGT GC	GTT GCC GAA AAT AGG GTC GC
Renilla luciferase	AGC GGG AAT GGC TCA TAT CG	CAA GCA CCA TTT TCT CGC CC
ORF11 gRNA 1	TGA ATG ACG GAA TCC CAT AC	
ORF11 gRNA 2	GTG GAG GAT CTG GCC GAC GC	

Supplementary Table 2. All qPCR and gRNA primers used in this study. Displayed as 5'-3'.

Target	Origin	Working Dilution	Supplier	Catologue #
GAPDH	Mouse	1:5000	Proteintech Europe	60004-1-Ig
FLAG	Rabbit	1:5000	Sigma-Aldrich	77425
		1:250 (IF)		
BUD23	Rabbit	1:500	Thermo Fisher Scientific	PA521698
		1:50 (IF)		
NOC4L	Rabbit	1:500	Proteintech Europe	17025-1-AP
eS19	Rabbit	1:500	Proteintech Europe	15085-1-AP
ORF57	Mouse	1:1000	Santa Cruz	sc-135747
CDK1	Mouse	1:5000	Abcam	ab18
ORF59	Rabbit	1:1000	A gift from Prof. Britt Glaunsinger (University of California, Berkeley)	N/A
K8.1	Mouse	1:1000	Advanced Biotechnologies	13-212-100
ORF65	Rabbit	1:500	Cambridge Research Biochemicals	crb2005224
GFP	Mouse	1:5000	Proteintech Europe	66002-1-Ig
DIMT1	Rabbit	1:500	Proteintech Europe	15563-1-AP
uS3	Rabbit	1:500	Proteintech Europe	11990-1-AP
uL23	Rabbit	1:500	Proteintech Europe	16386-1-AP
LANA	Rat	1:50 (IF)	Sigma-Aldrich	MABE1109
V5	Mouse	1:1000	Abcam	ab27671

Supplementary Table 3. All primary antibodies used in this study for western blotting and immunofluorescence (IF).
