# Supplementary material for: Kaposi’s sarcoma-associated herpesvirus induces specialised ribosomes to efficiently translate viral lytic mRNAs
Source: Nat Commun. 2023 Jan 18;14:300. doi: 10.1038/s41467-023-35914-5 (PMC9849454; doi:10.1038/s41467-023-35914-5)
Supplement: Supplementary file 3 — Description of Additional Supplementary Files [file 41467_2023_35914_MOESM3_ESM.pdf]

Description of Additional Supplementary Files

Supplementary Data 1. Processed mass spectrometry data for TREx BCBL1-Rta cell lines expressing FLAG-2xStrep tagged ribosome biogenesis bait protein pulldowns.

Cell lysates of latent and lytically reactivated control or TREx BCBL1-Rta cell lines expressing FLAG-2xStrep tagged ribosome biogenesis bait proteins (PNO1, LTV1, DMT1 and TSR1) were incubated with Magnetic Strep-TactinXT coated beads. The beads were then analyzed using tandem mass tagging (TMT) coupled to liquid chromatography (LC), mass spectrometry (MS) analysis. For each sample the background abundance values for each protein from the control TREx BCBL1-Rta cell lysate pulldown were taken away from the abundance value of each protein identified in the FLAG-2xStrep tagged ribosome biogenesis bait protein sample. A cutoff abundance value of 150 was then selected and all proteins with a lower value were discarded from further analysis.

Supplementary Data 2. Processed mass spectrometry data for TREx BCBL1-Rta cell lines expressing KSHV ORF11-GFP protein pulldowns.

Cell lysates of latent and lytically reactivated control or TREx BCBL1-Rta cell lines expressing ORF11-GFP were incubated GFP-trap affinity beads. The beads were then analyzed using tandem mass tagging (TMT) coupled to liquid chromatography (LC), mass spectrometry (MS) analysis. For each sample the background abundance values for each protein from the control GFP only pulldown were taken away from the abundance value of each protein identified in the GFP-ORF11 pulldown sample. A cutoff abundance of >1% of each protein compared to the GFP-ORF11 bait and >1.5 fold enrichment over the control GFP only pulldown.
