# Supplementary material for: Kaposi’s sarcoma-associated herpesvirus induces specialised ribosomes to efficiently translate viral lytic mRNAs
Source: Nat Commun. 2023 Jan 18;14:300. doi: 10.1038/s41467-023-35914-5 (PMC9849454; doi:10.1038/s41467-023-35914-5)
Supplement: Supplementary file 6 — Reporting Summary [file 41467_2023_35914_MOESM6_ESM.pdf]

Corresponding author(s): Adrian Whitehouse

Last updated by author(s): 12/14/2022

Reporting Summary

Nature Portfolio wishes to improve the reproducibility of the work that we publish. This form provides structure for consistency and transparency in reporting. For further information on Nature Portfolio policies, see our Editorial Policies and the Editorial Policy Checklist.

Statistics

For all statistical analyses, confirm that the following items are present in the figure legend, table legend, main text, or Methods section.

n/a	Confirmed	
☐	☒	The exact sample size (*n*) for each experimental group/condition, given as a discrete number and unit of measurement
☐	☒	A statement on whether measurements were taken from distinct samples or whether the same sample was measured repeatedly
☐	☒	The statistical test(s) used AND whether they are one- or two-sided<br>*Only common tests should be described solely by name; describe more complex techniques in the Methods section.*
☐	☒	A description of all covariates tested
☒	☐	A description of any assumptions or corrections, such as tests of normality and adjustment for multiple comparisons
☐	☒	A full description of the statistical parameters including central tendency (e.g. means) or other basic estimates (e.g. regression coefficient) AND variation (e.g. standard deviation) or associated estimates of uncertainty (e.g. confidence intervals)
☐	☒	For null hypothesis testing, the test statistic (e.g. *F*, *t*, *r*) with confidence intervals, effect sizes, degrees of freedom and *P* value noted<br>*Give P values as exact values whenever suitable.*
☒	☐	For Bayesian analysis, information on the choice of priors and Markov chain Monte Carlo settings
☒	☐	For hierarchical and complex designs, identification of the appropriate level for tests and full reporting of outcomes
☒	☐	Estimates of effect sizes (e.g. Cohen's *d*, Pearson's *r*), indicating how they were calculated

Our web collection on statistics for biologists contains articles on many of the points above.

Software and code

Policy information about availability of computer code

Data collection	No software was used
Data analysis	Graphpad Prism 9 calculator<br>TIDE: Tracking of Indels by DEcomposition - Version 3.3.0<br>FastQC Version 0.11.9<br>Cutadapt (v3.2)<br>Bowtie2 v.2.3.4.2<br>Samtools v1.9<br>RiboRev Version 2.4.0)

For manuscripts utilizing custom algorithms or software that are central to the research but not yet described in published literature, software must be made available to editors and reviewers. We strongly encourage code deposition in a community repository (e.g. GitHub). See the Nature Portfolio guidelines for submitting code & software for further information.

Data

Policy information about availability of data

All manuscripts must include a data availability statement. This statement should provide the following information, where applicable:

- Accession codes, unique identifiers, or web links for publicly available datasets
- A description of any restrictions on data availability
- For clinical datasets or third party data, please ensure that the statement adheres to our policy

Quantitative mass spectrometry datasets have been deposited to the PRIDE, Proteomics Identifications Database and are publically available under project accession codes PXD032318 (http://www.ebi.ac.uk/pride/archive/projects/PXD032318) for Pre-40S affinity purification proteomics and PXD032367 (http://www.ebi.ac.uk/pride/archive/projects/PXD032367) for KSHV ORF11 affinity purification proteomics. The Poly-ribo-seq dataset has been deposited to NCBI GEO, Gene Expression Omnibus and is publically available under GEO accession code GSE199095 (https://www.ncbi.nlm.nih.gov/geo/query/acc.cgi?acc=GSE199095). Other publically available datasets used in this analysis include Human hg38 rRNAs (Gencode v36) (https://www.gencodegenes.org/human/release_36.html) and tRNA sequences (GtRNAdb 18.1) (http://gtrnadb.ucsc.edu/archives.html).

Human research participants

Policy information about studies involving human research participants and Sex and Gender in Research.

Reporting on sex and gender	N/A
Population characteristics	N/A
Recruitment	N/A
Ethics oversight	N/A

Note that full information on the approval of the study protocol must also be provided in the manuscript.

Field-specific reporting

Please select the one below that is the best fit for your research. If you are not sure, read the appropriate sections before making your selection.

☒ Life sciences ☐ Behavioural & social sciences ☐ Ecological, evolutionary & environmental sciences

For a reference copy of the document with all sections, see nature.com/documents/nr-reporting-summary-flat.pdf

Life sciences study design

All studies must disclose on these points even when the disclosure is negative.

Sample size	No sample size calculation was conducted. We selected samples sizes of sufficient size to ensure reproducibility of our findings as well as large enough to perform statistical analyses (at least n=3 unless stated otherwise). Our rational for selecting this sample size was primarily based on previous experience with these assays informing us on the number of replicates required to achieve statistical significance given the variance of each assay.
Data exclusions	No data were excluded from this study. We pre-established our exclusion criteria as a rejection of a dataset if either our positive controls or negative controls failed.
Replication	We repeated experiments at least three times or the number indicated in the figures legends.
Randomization	Experiments were not randomized. Data variability was controlled through the inclusion of multiple biological replicates, inclusion of multiple technical replicates within an experiment, and utilization of distinct guide RNAs targeting a single gene in multiple cell lines.
Blinding	Researchers were not blinded during experiments which was a result of working with KSHV and the high complexity of the conducted experiments.

Reporting for specific materials, systems and methods

We require information from authors about some types of materials, experimental systems and methods used in many studies. Here, indicate whether each material, system or method listed is relevant to your study. If you are not sure if a list item applies to your research, read the appropriate section before selecting a response.

Materials & experimental systems

n/a	Involved in the study
☐	☒ Antibodies
☐	☒ Eukaryotic cell lines
☒	☐ Palaeontology and archaeology
☒	☐ Animals and other organisms
☒	☐ Clinical data
☒	☐ Dual use research of concern

Methods

n/a	Involved in the study
☒	☐ ChIP-seq
☒	☐ Flow cytometry
☒	☐ MRI-based neuroimaging

Antibodies

Antibodies used

The following antibodies were used in this study dilutions shown are for western blotting unless stated otherwise. All secondary antibodies were used at a concentration of 1:5000.

GAPDH Mouse 1:5000: Proteintech Europe, 60004-1-Ig
FLAG Rabbit 1:5000 for WB and 1:250 IF: Sigma-Aldrich, 774251:250
BUD23 Rabbit 1:500: for WB and 1:50 IF: Thermo Fisher Scientific ,PA521698
NOC4L Rabbit 1:500: Proteintech Europe, 17025-1-AP
eS19 Rabbit 1:500: Proteintech Europe ,15085-1-AP
ORF57 Mouse 1:1000: Santa Cruz, sc-135747
CDK1 Mouse 1:5000 :Abcam, ab18
ORF59 Rabbit 1:1000: A gift from Prof. Britt Glaunsinger (University of California, Berkeley) N/A
K8.1 Mouse 1:1000: Advanced Biotechnologies, 13-212-100
ORF65 Rabbit 1:500: Cambridge Research Biochemicals, crb2005224
GFP Mouse 1:5000: Proteintech Europe, 66002-1-Ig
DIMT1 Rabbit 1:500: Proteintech Europe, 15563-1-AP
uS3 Rabbit 1:500: Proteintech Europe, 11990-1-AP
uL23 Rabbit 1:500 : Proteintech Europe, 16386-1-AP
LANA Rat 1:50 (IF): Sigma-Aldrich, MABE1109
V5 Mouse 1:1000: Abcam, ab27671

Validation

Each antibody was validated using cellular lysates from both positive and negative controls (parental vs. KO cells). Only antibodies that validated according to the manufactures instructions or utilised in previously published articles were used in this study.

Eukaryotic cell lines

Policy information about cell lines and Sex and Gender in Research

Cell line source(s)

TREx BCBL1-Rta cells, a primary effusion lymphoma B cell line latently infected with KSHV and modified to contain doxycycline inducible myc-RTA, were a kind gift of Jae U. Jung (University of Southern California.
HEK-293T cells (American Type Culture Collection)

Authentication

Cells came authenticated from ATCC. TREx BCBL1-Rta cells were routinely screened for KSHV reactivation using IFA using LANA and ORF57 specific markers,

Mycoplasma contamination

All cells lines tested negative for mycoplasma and are routinely screened.

Commonly misidentified lines
(See ICLAC register)

HEK-293T
